# Laparoendoscopic single site surgery in pediatric urology: does it require specialized tools?

**DOI:** 10.1590/S1677-5538.IBJU.2014.0444

**Published:** 2016

**Authors:** Nishant Patel, Michael Santomauro, Sarah Marietti, George Chiang

**Affiliations:** 1 Department of Urology, University of California San Diego Health System, San Diego, California; 2 Institute of Urology, University of Southern California, Los Angeles, California; 3 UCSD Department of Urology, Rady Children's Specialists Pediatric Urology, San Diego, California

**Keywords:** Laparoscopy, Pediatrics, Surgical Procedures, Operative, Urology

## Abstract

**Purpose:**

To describe our experience utilizing Laparoendoscopic single site (LESS) surgery in pediatric urology.

**Materials and Methods:**

Retrospective chart review was performed on LESS urologic procedures from November 2009 through March 2013. A total of 44 patients underwent 54 procedures including: nephrectomy (23), orchiopexy (14), varicocelectomy (9), orchiectomy (2), urachal cyst excision (3), and antegrade continence enema (3) (ACE).

**Results:**

Median patient age was 6.9 years old. Estimated blood loss (EBL), ranged from less than 5cc to 47cc for a bilateral nephrectomy. Operative time varied from 56 mins for varicocelectomy to a median of 360 minutes for a bilateral nephroureterectomy. Incision length ranged between 2 and 2.5cm. In our initial experience we used a commercial port. However, as we progressed, we were able to perform the majority of our procedures via adjacent fascial punctures for instrumentation at the single incision site. One patient did require conversion to an open procedure as a result of bleeding. Three complications were noted (6.8%), with two Clavien Grade 3b complications. Two patients required additional procedures at 1-year follow-up.

**Conclusions:**

The use of LESS applies to many pediatric urologic procedures, ideally for ablative procedures or simple reconstructive efforts. The use of adjacent fascial puncture sites for instrumentation can obviate the need for a commercial port or multiple trocars.

## INTRODUCTION

With the advent of minimal invasive surgery, urology has moved to the forefront in regards to innovation and instrumentation in its use compared to open surgery without jeopardizing functional outcomes. With increasing experience in the laparoscopic environment, efforts are now directed at further minimizing the number of incisions while maintaining the basics tenets of laparoscopic surgery (1). This focus has led surgeons to perform traditional laparoscopic surgery through a single incision. LESS is an evolution of minimal access surgery that promises virtually scarless abdominal operations (2). Although several variants of this approach have been reported, a convened international multi-disciplinary consortium of experts have coined the term LESS (Laparoendoscopic Single Site) surgery to collectively encompass laparoscopic procedures performed through a single skin incision (3).

As LESS has proliferated in the adult population, many LESS techniques are starting to permeate the pediatric population as well. There have been a variety of descriptions on the approach to LESS including the use of conventional versus articulating instruments as well as commercial ports (4). This advancement and transformation to the pediatric population is starting to gain momentum as an option for surgical intervention with overall cosmesis as a surrogate outcome marker (5). We report on our 3-year experience of LESS in children who underwent a variety of urologic procedures utilizing this technique.

## MATERIALS AND METHODS

**Figure 1:**
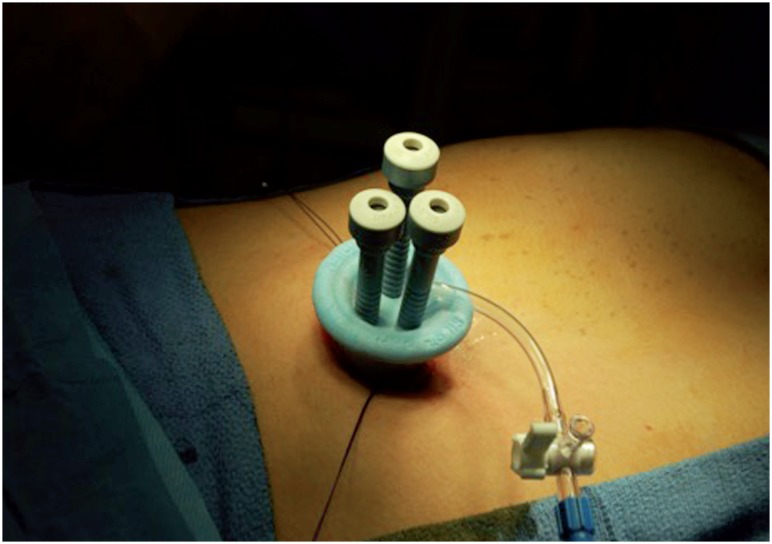
SILS(tm) port in use at the umbilicus, which was utilizes for the majority of LESS procedures.

After Institutional Review Board approval, a retrospective chart review was performed of all patients who underwent a planned LESS procedure from November 2009 through March 2013 at Rady Children's Hospital by two surgeons with previous traditional laparoscopic experience (GC, SMS). Inclusion criteria included all patients being seen for consideration of nephrectomy, varicocelectomy, antegrade continence enema (ACE), urachal cyst excision, intra-abdominal orchiopexy or orchiectomy. Decision for LESS versus a conventional laparoscopic approach was dictated by surgeon preference and experience. Informed consent was obtained by the parents/guardians in describing the difference between standard laparoscopy and LESS at the time of their pre-operative appointment. Outcome measures included operative time, estimated blood loss, hospital stay, peri-operative complications, inpatient narcotic doses when applicable, pain scores (FACES or verbal 1-10), conversions to open or standard laparoscopy, and the need for a second subsequent procedure. Initially all nephrectomies were performed using the Covidien SILS^™^ port (Dublin, Ireland). Our technique has been previously reported (Figure-1) (3, 6).

### Surgical technique

**Figure 2:**
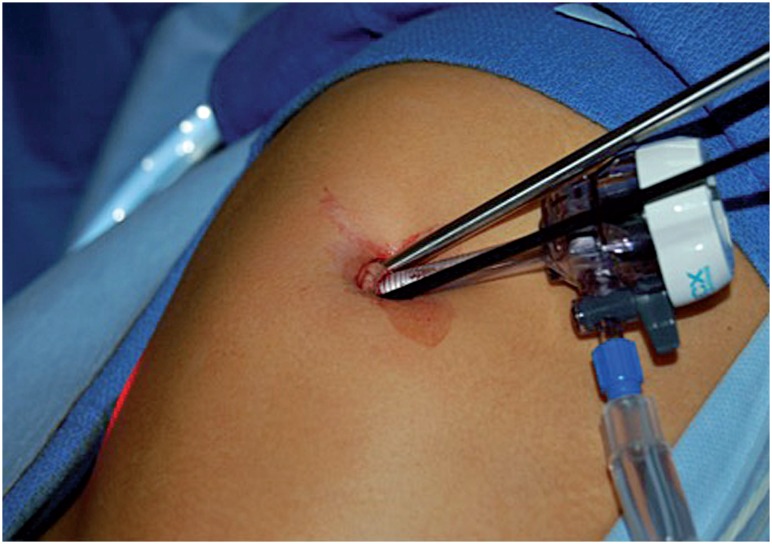
Modified LESS technique used traditionally for appendectomy.

Recently we have adapted an approach utilized by general surgeons for appendectomies in non-nephrectomy procedures. After a 2 to 2.5cm longitudinal or transverse skin incision through the umbilicus, pneumoperitoneum was performed utilizing a Veress needle via the umbilical ring. A 5mm trocar was then placed under direct vision through the umbilical ring. A#11 blade was then used to create 2 small fascial stab incisions above and below or lateral to the 5mm trocar depending on the planned procedure. A 3mm grasper and a 45º bariatric length telescope with a right angle light cord adaptor were placed via the fascial incisions, obviating the need for a multiport (Figure-2). The grasper and the telescope remained intracorporeal throughout the procedure. Cleaning of the laparoscope was performed as needed against the anterior abdominal wall. A SILS port was utilized for all nephrectomies. Our single incision fascial puncture technique was used for all other groin procedures.

## RESULTS

A population of 44 patients underwent a total of 54 procedures. The extent of the procedures included 23 nephrectomies, 14 orchiopexies, 9 varicocelectomies, 2 orchiectomy/gonadectomies, 3 urachal cyst excisions and 3 antegrade continence enema (ACE). Table-1 displays the descriptive data for our LESS experience. Some patients required additional procedures (i.e circumcision, Deflux injection) at the same setting. The median age was 6.9 years old (range 7 months to 18 years old). The operative time varied based on the procedure performed, from 56 minutes for a varicocelectomy to an average of 345 minutes for a bilateral nephroureterectomy. All non-nephrectomy cases were performed without a commercial port using our single incision fascial puncture technique. The indications for unilateral nephrectomy were malfunctioning kidney secondary to multicystic dysplastic kidney (38%), vesicoureteral reflux (23%), ureterocele (15%), and ureteropelvic junction obstruction (23%). The indications for bilateral nephoureterectomy were Denys-Drash syndrome (20%), posterior urethral valves (20%), and medical renal disease (60%). The median pain score (FACES or verbal) ranged from 1.1 to 1.9 and the median number of morphine equivalent doses (oral, intravenous) ranged from 1 to 6.5. The median length of stay ranged from 0.87-5.2 days.

**Table 1:** Operative Data for Pediatric Urologic LESS Procedures

Procedure Type	Number of Patients	LESS approach	Median Operative Time (mins), IQR	Median EBL (cc), IQR	Other procedures during surgery	Median Length of Stay (days), IQR	Median Pain Score POD 1 (1-10), IQR	Median morphine equivalent doses POD1, IQR	Complications	Need for additional operation
Unilateral Nephrectomy	13	Port	178,65	10,5	1 patient with circumcision	2.1, 1.5	1.8, 1.4	5,3	1 patient with Ileus requiring PICC line and TPN 1 patient with wound infection	1 patient with PICC line under anesthesia
Bilateral Nephroureterectomy	5	Port	360, 106	50, 58	1 patient with gonadectomy 1 patient with circumcision /cystoscopy	5.2, 1.5	1.9, 3.1	6.5, 3.5		
Varicocelectomy	9	Adjacent fascial stab Incisions	85, 34	5, 0		N/A	N/A	N/A		1 patient required a subsequent IR embolization for persistence
Orchiopexy	11	Adjacent fascial stab Incisions	89, 41	5, 0	2 patients with orchiectomy 2 patient with BL orchiopexy 1 patient with BL orchiopexy and circumcision	N/A	N/A	N/A		
Urachal cyst excision	3	Adjacent fascial stab Incisions	87, 17	5, 0		0.87, 0.04	1.1, 0.5	1,1		1 patient required an open operation for remnant removal 10 months post-op
ACE	3	Adjacent fascial stab Incisions	109, 42	15, 10	1 patient with bilateral Deflux injection	2.1, 0.1	1.5, 0.7	3,3	1 patient with ACE stenosis	This patient required colonoscopy and catheter placement and subsequent Chait tube placement both under general anesthesia

**IQR=** Interquartile Range, **EBL=** Estimated blood loss, **PICC=** peripherally Inserted central catheter, **TPN=** total parenteral nutrition, **IR=** Interventional radiology, **BL**=bilateral, **ACE=** antegrade continence enem

Despite the fact that the procedures varied in purpose, the incision length ranged between 2 and 2.5cm for all the procedures. This size incision was appropriate for the commercial multiport as well as the described fascial stab incision approach. We report 3 complications (6.8%) including two Clavien 3b complications. After unilateral nephrectomy, one patient developed ileus, required a peripherally inserted central catheter (PICC) inserted under general anesthesia and initiation of total parenteral nutrition (TPN). Another patient developed stenosis of her ACE and required two additional operations under general anesthesia-colonoscopy and catheter placement, then subsequent Chait tube placement. One patient developed a Clavien 1 wound infection after unilateral nephrectomy, which was treated with drainage in the office and antibiotics. Early in our series one patient did require conversion to an open procedure secondary to bleeding at the hilum during unilateral nephrectomy, however, no transfusion was required. In regards to long-term follow-up, 1 patient required subsequent embolization of his gonadal vein secondary to varicocele persistence and another patient required an additional operation to remove a remnant of a urachal cyst.

## DISCUSSION

The first laparoscopic pediatric nephrectomy was described in 1992 (7) but over the last decade, urology has witnessed an exponential increase in laparoscopic and robotic surgery for the treatment of various surgical disorders (1), with the first pediatric LESS nephrectomy being reported in 2009 (8). Standard laparoscopy results in less incisional morbidity, less narcotic usage, and shorter hospital stay with equivocal functional outcomes when compared to open surgery (9, 10). To optimize the benefit of minimally invasive surgery, surgeons have attempted to reduce the overall abdominal wall incision by decreasing either the size or the numbers of trocars used during procedures (11). Minimizing the incisions without compromising intracorporeal access lends to the advantages of LESS in that the entire operation can be done using an incision that would have been necessary for specimen removal in conventional laparoscopy. An entire operation can be performed through an approximately 2cm incision.

Our report details our initial series of 54 LESS urologic procedures for various indications in the pediatric population. Treatment efficacy, in this early experience and follow-up seems to approach those procedures performed by standard laparoscopy. Other centers have confirmed the applicability of LESS in pediatric urology (8, 12–14). In our experience, although 1 patient required conversion to an open procedure, alternating to conventional laparoscopy would not have changed the eventual surgical course. Our complications included post-operative ileus, post-operative superficial wound infection and return to the operating room for stenosis of an antegrade continence enema. Two other patients required additional procedures including an embolization for a persistent varicocele and an open re-excision of an urachal cyst remnant. We do not feel that a conventional laparoscopic approach would have yielded a different initial result. In regards to pain medication and hospital stay, our prior experience demonstrated that LESS did not significantly change their postoperative course in regards to postoperative pain scores, length of stay, or use of post operative narcotics as compared to our standard laparoscopy patients (3, 6).

With our LESS technique, there were obvious technical challenges encountered. These stem from the inherent nature of LESS including loss of triangulation, difficulty retracting, instrument crowding and crossing, and in-line vision to name a few (11). Even with considerable laparoscopic experience, the learning curve in performing LESS is fairly steep and requires considerable patience and time.

Despite the inherent challenges, we found that LESS was possible in the pediatric population using standard pediatric instrumentation. We did not require use of articulating instruments and most recently we have avoided use of a commercial multiport while reducing the number of trocars used. A port is still used for both unilateral and bilateral nephrectomies owing to the need for exchange of multiple instruments, but all groin cases and ACE procedures can be performed simply with one central trocar and 2 adjacent fascial stab incisions for the telescope and a grasper. We have found that positioning the grasper on the side closest to the organ of interest is ideal with the telescope on the opposite side. Occasionally a 2.3mm percutaneous MiniLap alligator grasper (Stryker, San Jose, CA) has been used during nephrectomies for retraction. This has been a criticism to the concept of single site surgery but the puncture wound is equivalent to an 11-gauge needle that requires only an adhesive strip for closure.

Some aids to limit the loss of triangulation and instrument clashing include: 1) The use of a 45 degree bariatric length telescope which provides the advantage of angulation and distance away from the hand instruments to prevent clashing. 2) The camera can be zoomed to offset the relative farsightedness that can become apparent inside the abdomen and. 3) Counter traction during dissection can be assisted with crossing of the standard laparoscopic instruments to allow additional retraction and angulation (3). 4) Retraction with a grasper should be performed prior to insertion of an instrument for actual dissection; this minimizes clashing and preserves exposure.

When looking at our technique and experience, we found that the LESS technique was ideal for ablative type procedures. Simple reconstructive procedures that do not require extensive intracorporeal suturing can also be performed safely and effectively. In the future as technology improves, various instrumentation may further assist and improve the efficiency of these operations and expand the possible indications.

With our current experience and prior study (3), we currently do not believe time to recovery nor narcotic usage is significantly decreased when compared to standard laparoscopy in regards to procedures requiring inpatient admission. However, we did not assess overall pain scores or narcotic usage for outpatient procedures (orchiopexy and varicocelectomy). Bansal, et al. demonstrated that LESS varicocelectomy patients required a higher number of narcotic doses in the recovery room compared to conventional laparoscopic varicocelectomy (15).

In theory, LESS and conventional laparoscopy for the same procedure should result in similar peri-operative outcomes. Tam et al. demonstrated no difference in postoperative analgesic requirement and hospital stay when comparing patients undergoing LESS nephrectomy versus conventional laparoscopic nephrectomy. The mean operative was significantly longer in the LESS group (156 mins versus 99 mins) (4). Dutta et al. reported on their single incision laparoscopic surgery experience on twenty patients. They concluded that single site surgery can be performed in children with outcomes similar to standard laparoscopic surgery, while affording outstanding cosmesis (2). With regard to long-term follow-up, two of the patients in our series required additional operative procedures-IR embolization for persistent varicocele and open operation for excision of urachal remnant. We do not believe these outcomes are a result of the LESS approach, as a known 4% recurrence rate occurs for standard laparoscopic varicocelectomy and urachal remnants are generally difficult to localize through any approach. The importance of cosmesis and outward physical appearance in regards to surgery remains as an unknown variable. The psychological impact of visible abdominal scarring needs further study, as it is the one consequence of a surgical procedure that persists long after pain has resolved and recovery is complete (2, 16).

Our series does not demonstrate an inferiority or superiority to conventional laparoscopy or open surgery. However, it does demonstrate a wide range of procedures that can be performed successfully in the pediatric urology patient. Our current position is to attempt LESS initially in the operations noted. We are not precluded from placing additional trocars or open conversion if required. Cosmetic outcomes may seem insignificant in terms of perceived surgical benefit, however, further studies may be required in measuring the psychological impact of physical scarring in children in their recovery from surgery.

## CONCLUSIONS

There continues to be a debate on the utility of LESS in all of adult and pediatric surgery. We have found it to be as safe and effective for ablative procedures or simple reconstruction. Over time, we have found that commercial ports are not necessary for short, simple procedures. In addition, specialized articulating instruments are not necessary for the procedures we have performed.

## Abbreviations

LESS: = Laparoendoscopic single site
ACE: = Antegrade Continence Enema
SILS: = Single Incision Laparoscopic Surgery
TPN: = Total Parenteral Nutrition
EBL: = Estimated Blood Loss
